# Serenity Integrated Mentoring and the High Intensity Network: a scheme that raises serious questions for practice and governance in UK psychiatry

**DOI:** 10.1192/bjb.2022.6

**Published:** 2023-02

**Authors:** Allan House

**Affiliations:** School of Medicine, University of Leeds, UK

**Keywords:** Suicide, crisis services, stigma and discrimination, service users, psychiatry and law

## Abstract

Serenity Integrated Mentoring (SIM) involved the police and mental health crisis services working in a single team, developing case management plans that allowed a seamless move from offers of therapeutic engagement (by the mental health team) to use of coercive measures (by the police) with those who persisted with frequent crisis presentations. Withdrawn after widespread criticism, the scheme raises important questions – about the practice of mental health professionals who are involved in decisions about using criminal sanctions for people presenting in crisis, about the ethical and legal status of the sharing of confidential clinical information with the police, and about the processes that professional bodies use in promoting, monitoring and responding to controversial service developments.

## What is Serenity Integrated Mentoring (SIM)?

The recently suspended High Intensity Network managed a form of police involvement in mental health crisis work known as SIM^a^. The SIM scheme, originally known as the Integrated Recovery Programme, involved staff from mental health services and the police working in a single team that developed and delivered a management plan to people identified as high intensity users of services. The focus of the scheme was people who had recurrent mental health crises – typically with suicidal thoughts or self-harm – that involved frequent contact with both the police and mental health crisis services. Such contacts were seen as counterproductive for all concerned, and the central idea was to develop a service model that made them less likely. The aim of SIM was to allow a seamless move from offers of therapeutic engagement (by the mental health team) to use of coercive measures (by the police) if deemed necessary with those who persisted with crisis presentations – such measures including community behaviour orders and prosecution for wasting police time.

The SIM programme was developed by a police officer on the Isle of Wight in 2013. An initial report^1^ into its effectiveness in eight people led to take-up and enthusiastic promotion by the local Academic Health Science Network (AHSN), and a number of awards followed. The SIM lead obtained an innovation fellowship and left the police to establish the High Intensity Network as a vehicle for extending and supporting the scheme. At one stage, it was reported that over half the mental health Trusts in England and Wales were involved.

## Criticism of the SIM scheme

Criticism of SIM came early from individuals with experience of it and was coordinated in a user-led group, the StopSIM coalition (https://stopsim.co.uk). Among the many issues raised were concerns about the damaging effects of punitive intervention by the police, application of the SIM process without full and free consent by service users, and sharing by mental health services of confidential clinical information with the police. It was noted that the two small evaluations^1,2^ undertaken had failed to provide convincing evidence of benefit or to make an adequate attempt at systematic identification of harms.

The High Intensity Network's literature used frankly offensive language at times,^3^ for example, noting that ‘The project team will be dealing with service users with often high risk, malicious and litigious behaviours’. It is difficult to read the Network's materials, with their references to ‘behavioural illness’ and emphasis on the link between mental disorder and crime, and avoid the conclusion that those who were the focus of the scheme were to be seen as trouble-makers in need of firm management: ‘Our teams operate multi-agency panels each month to assess which patients should have their consent removed …’. Complaints were framed as ‘allegations’, and it was suggested that staff dealing with them should first watch a video produced by the High Intensity Network and respond accordingly, rather than simply following usual Trust complaints procedures.

The approach to sharing of confidential clinical data was similarly authoritarian, noting that crisis plans might be developed by sharing clinical information in the team at any time, because patients in the scheme ‘are constantly an emergency case’. At the same time, clinical staff were advised about these plans ‘please don't tell the patient they have any right to choose who reads it. Know your GDPR’.

Professional bodies were slow to pick up the issue. Strong reservations were eventually expressed in 2021 by the Centre for Mental Health^4^ and the British Association of Social Workers (BASW),^5^ and in May a letter was sent by Tim Kendall (the National Clinical Director of NHS England) to all medical directors of mental health Trusts, asking them to review their involvement with the scheme, including the ethical and legal basis for sharing information with the police.

In response to such questioning, the director of the High Intensity Network reduced a previously high-profile online presence, for example, deleting his Twitter and LinkedIn accounts. The last straw seems to have been the emergence of responses to a Freedom of Information request, which indicated that the Hampshire police had previously raised concerns about misrepresentation of police data in the initial reports of the scheme's effectiveness and raised other concerns about misrepresentation in the way the scheme was being promoted. In an unannounced move, the High Intensity Network website has now been taken down and the director of the programme is incommunicado.

## The College's response

In May 2021 the College announced that it was investigating the scheme in the light of these concerns, articles about which had also appeared in the national press.^6,7^ The College has more recently issued a statement^8^ acknowledging that something has been seriously amiss about the implementation of SIM and about the slowness of professional responses.

The College has held a number of discussions of the issue with the central NHS England and Innovation (NHSE/I) mental health team, pushing for them to ensure that the reviews they asked Trusts to conduct are done quickly and robustly. The College has raised questions about how and why (given the lack of evaluation of patient outcomes) the SIM programme was rolled out from the pilot phase and what steps were taken to follow up concerns about misuse of data.

In the longer term, the College hopes to work with NHSE/I to agree on a common, evidence-based approach to best supporting people who might be described as high intensity users of crisis services in England, informed by the College's Faculties and including input from those with direct personal experience. No timetable has been set for this work, and the form and content of any review has yet to be specified.

## Looking ahead – what needs to happen next?

The affair cannot be left there – it raises important questions about culture and practice in psychiatry, and at an organisational level about governance when clinical services move into new ways of working. The specific SIM scheme managed by the High Intensity Network may be defunct, but it is understood by the College that many Trusts operate their own similar versions. One likely reason is that government austerity policies have led to years of underfunding of mental health services^9^ and of the police at a time of increased pressure due to a deterioration in community resources, creating circumstances in which a culture of intolerance of perceived excessive service use can readily develop.

The College needs to be supported to deliver timely and specific recommendations. There are at least three areas that need urgent attention from the next round of inquiry. The questions raised are relevant for future practice at the interface between health services and the criminal justice system: answering them should be the responsibility not just of NHS England but of the College itself, because they bear directly on the clinical practice of its members. It is disconcerting that the scheme received the professional support it did, including endorsement by national figures in psychiatry. For such schemes to run at all there must have been acceptance among participating local clinicians (including medical directors) that it is justified to support coercive and criminalising responses to so-called high intensity crisis contact – even though such contact might not be remotely considered dangerous and even though there is no evidence of benefit to patients from such approaches. It is no doubt relevant that many involved patients will have been given a diagnosis of personality disorder – a diagnosis which can lead to failure to offer consistent care to people who are distressed and at risk of suicide.^10,11^

The first question relates to psychiatric practice in situations where the police are involved in mental health crises in the community, such as in street triage services and Section 136 contacts. Primarily from the point of view of the well-being of individuals and also from that of the standing of psychiatry (examples of negative press comments are provided in the references^12^) it is important to limit coercive interventions to the absolute minimum compatible with the safety of all concerned.^13^ In relation to involvement of the police in crisis management, the College should offer clear criteria that go beyond general references to the need for the bar to be set higher than it was by SIM. Most pressing is a need for the College to make a clear statement about whether it thinks there is *ever* a justification for mental health professionals to be involved in decisions about criminal sanctions for people presenting in crisis and, if so, under what circumstances.

Second, clinicians need more specific direction about the ethical and legal status of the practice of sharing confidential clinical information with the police. Again, the threshold was clearly set far too low by SIM, and the associated misrepresentation of the principles has muddied the waters. There is a question to be answered about the specific circumstances in mental health crisis work under which either vital interests or public task justifications can be used to share unconsented data.^14^

Third, the College needs to rethink its current position in relation to people given a diagnosis of personality disorder. The latest College statement^15^ is framed in a way that suggests those who dislike the diagnosis are part of a problem to be resolved by further education and by provision of more personality-disorder-specific services. History does not suggest that such an approach will reduce negative stereotyping and related exclusionary practices; what is needed is a move to more tolerant approaches to dissent from use of the diagnosis, coupled with the provision of patient-centred services based on need rather than diagnosis.

As part of these actions, as recognised by the College, there should be strengthening of the processes used in monitoring and responding to service developments that arouse strong reactions from patients and other service users. It is a sign of deficiency in current systems that so much of the pressure to review this scheme had to be applied through social media and related campaigns, and, to quote the College statement ‘any review must examine why professional frameworks did not identify or act on these concerns’. A question that should of course be answered in collaboration with relevant patient groups.

Answers to some of these questions cannot await an extended review. New National Institute for Health and Care Excellence guidelines on the management of self-harm, now out in draft form for consultation,^16^ say unequivocally ‘Do not use aversive treatment, punitive approaches or criminal justice approaches such as community protection notices, criminal behaviour orders or prosecution for high service use as an intervention for frequent self-harm episodes’. In a social media post,^17^ Tim Kendall has already expressed his support for that view. We therefore need a statement from the College sooner rather than later.

The SIM scheme sheds light on some of the most important challenges facing current psychiatry in the UK – how to manage the balance between pressures for innovation in the National Health Service against those for evidence-based and patient-centred practice; ensuring effective collaborative working with agencies such as the police without being drawn into undesirable coercion and criminalisation of people in distress; and maintaining the balance between productive data-sharing to improve continuity and consistency of care and the need to respect patient confidentiality even in the most difficult of circumstances. It is for these reasons that further inquiry into the High Intensity Network/SIM debacle is, notwithstanding its demise, so badly needed.

## Footnotes

a Primary sources on SIM and the High Intensity Network have been mainly accessible online and have now all been withdrawn by the director. Secondary sources can be found in the reference list and by searching social media – for example, using the Twitter tags #StopSIM and #HighIntensityNetwork.

## About the author

**Allan House**, MRCPsych, DM, is Emeritus Professor of Liaison Psychiatry at the University of Leeds, UK.
