# Serenity Integrated Mentoring and the High Intensity Network: A scheme that raises serious questions for practice and governance in UK psychiatry – CORRIGENDUM

**DOI:** 10.1192/bjb.2022.20

**Published:** 2023-02

**Authors:** Allan House

**Keywords:** Suicide, crisis services, stigma and discrimination, service users, psychiatry and law

The author would like to correct two errors in the above article^1^. There was an incorrect word published in the title and abstract:

‘Serenity Integrated Monitoring’ should be ‘Serenity Integrated Mentoring’. This has since been updated in the online PDF and HTML versions.

The first heading ‘What is Serenity Integrated Monitoring (SIM)’ should also read ‘What is Serenity Integrated Mentoring (SIM)’.

There is a spelling error in a name in the Acknowledgments section. This should read Alex Thomson, not Alex Thompson. The author apologises for these errors.
